# Child and adolescent psychiatry services in Singapore

**DOI:** 10.1186/s13034-015-0037-8

**Published:** 2015-05-13

**Authors:** Choon Guan Lim, Say How Ong, Chee Hon Chin, Daniel Shuen Sheng Fung

**Affiliations:** Department of Child and Adolescent Psychiatry, Institute of Mental Health, 10, Buangkok View, 539747 Singapore, Singapore; Department of Psychological Medicine, KK Women’s and Children’s Hospital, Singapore, Singapore

**Keywords:** Child psychiatry, Child mental health, Adolescent psychiatry, Singapore, Education

## Abstract

Singapore is a small young city state with a multi-ethnic and multi-cultural population. This article reviews the development of the country’s child and adolescent psychiatry services through the years, in the background of other developments within the country’s education, social and legal services. Research and other available data on the prevalence of psychiatric problems among children and adolescents in Singapore are summarized, although there has been no nation-wide epidemiological study done. One of the most recent developments has been the establishment of a community mental health service, which works collaboratively with schools and community partners. Some challenges are also discussed especially in the area of child and adolescent psychiatry training. Possible future directions include providing mental heath care for preschool children as well as epidemiological studies to identify disease prevalence and mental health needs among children and adolescents in Singapore.

## Introduction

Singapore is a small island located within Southeast Asia at the southern tip of the Malaysian Peninsula. We will provide a brief history of the country’s development to provide the demographic and social backdrop against which child and adolescent psychiatric services subsequently developed.

Following the arrival of Sir Stamford Raffles in 1819, Singapore transformed from a fishing village to become a flourishing British colony. In 1963, Singapore became part of the formation of Malaysia before gaining independence two years later. Singapore’s population is multi-ethnic, with Chinese making up the majority at 74%, followed by Malays (13%), Indians (9%) and other minorities (3%), reflecting the migrant origins of the resident population from the region. About 16% of the 3.8 million residents are under the age of 15 years [1]. Following decades of rapid development and economic growth, the country is almost 100% urbanized today with a land area of 716 sq km and a population of 5.4 million.

The country’s economic progress and the government’s policies had largely influenced population growth. A series of population control measures were implemented since the sixties, including the successful ‘Stop at Two’ policy, to avoid burdening the developing economy with an excessively large population. By 1986, the government reversed its policies to encourage childbirth because of falling birth rates and an aging population. In 2011, Singapore’s total fertility rate stood at a nadir of 1.20, way below the replacement rate of 2.1, continuing the trend over more than three decades [2]. Over the past decade, the divorce rate has also increased from about 1.2 to 1.9 divorces per 1000 residents. Table 1 shows some other demographic indices of Singapore residents.

**Table 1:** **Demographic indices of Singapore residents**

		**2006**	**2010**	**2013**
Total population (million)		4.4	5.1	5.4
Singapore residents (million)		3.5	3.8	3.8
Total				
Age:	% below 15 years	19.5	17.4	16.0
	% 15–64 years	72.2	73.6	73.5
	% 65 years & above	8.3	9.0	10.5
Total fertility rate (per female)		1.28	1.15	1.19
Crude birth rate (per 1,000 population)		10.3	9.3	9.3
Infant mortality rate (per 1,000 live-births)		2.6	2.0	2.0
Life expectancy at birth (years)		80.3	81.7	82.5
Male		77.8	79.2	80.2
Female		82.6	84.0	84.6

Since October 1995, Singapore has ratified the United Nations Convention on the Rights of the Child, setting minimum standards that governments should meet in providing legal, social and education services for children. Education was an early area of focus for the government as it recognized the need to build and train its working force in order for the country to develop economically. Although Malay is the country’s national language, English is the main medium of instruction and is taught as a first language in school. It was also mandated that students were educated in their mother tongue as a second language in school, either Chinese, Malay or Tamil. Children typically start preschool at age 3 and receive 2 years of nursery education and 2 years of kindergarten. Subsequent mainstream education includes 6 years of primary school and 4 to 5 years of secondary school. Tertiary education options include junior college (or pre-university), polytechnic and Institute of Technical Education. The Ministry of Education enacted the Compulsory Education Act in 2000 to make education compulsory for children of primary school age without disabilities to attend school, unless they have been granted permission for either homeschooling or attendance at a full-time religious institution. All 369 mainstream schools are staffed with counsellors and allied educators to provide additional support for children with emotional, behavioural or learning difficulties. There are also 21 special schools for children with disabilities, including intellectual disability, autism and physical disabilities, among others.

To help bring together organizations and individuals with common interest in community service and social welfare, the Singapore Council of Social Service was formed in 1958, before its restructuring to become the National Council of Social Service. Other than providing child protection services, the Ministry of Social and Family Development (MSF) enforces legislations on child welfare and protection cases, in addition to policy making on issues such as adoption, child care and education, abuse and violence, and people with disabilities. Laws which provide for welfare, care, protection and rehabilitation of young persons are clustered under the Children and Young Persons Act. Previous surveys conducted by MSF suggest that families remain a strong source of support for Singaporeans. MSF also oversees a network of Family Service Centres throughout the island to provide help for families in need.

Healthcare in Singapore is provided by both public and private sectors. There are a total of 8 public hospitals comprising 6 acute general hospitals, a women’s and children’s hospital (KK Women’s and Children’s Hospital) and a psychiatric hospital (Institute of Mental Health). Public primary healthcare is provided by a network of polyclinics across the island. The private sector similarly provides both primary and specialist care. Preschool mental health services are provided by primary care physicians and developmental pediatricians. Psychiatrists are based in tertiary hospitals and attend to individuals with more severe emotional and behavioural concerns. Every child is issued a health booklet at birth which records important health related information, vaccination history and developmental screening findings [3]. Developmental screening can be conducted by primary care doctors, usually conveniently timed with the immunisation schedule. If there is a need for further assessment when a developmental delay is identified, the child is referred to hospital- based child development units. A study of such cases referred to KK Women’s and Children’s Hospital (KKWCH), Singapore’s largest provider of paediatric services, showed that the most common presenting concern was speech and language delay, and that the most common diagnosis among these children was autism spectrum disorder [4].

### Prevalence of mental disorders in the young

Although national mental health surveys have been conducted to assess the prevalence of mental health disorders among adults and the elderly, there have been no similar national studies for the young. There is also little comparative data due to the lack of epidemiological data from our neighbouring countries and the dissimilar ethnic make-up among the Southeast Asian nations. The Singapore Mental Health survey of 2010 for adults aged 18 and above showed that many mental health disorders have their onset in childhood [5]. The prevalence of preschool mental health disorders was estimated in a clinic-based study to be at 7% [6]. In the only community based prevalence study involving 2139 school-going children aged 6–12 years, the prevalence of emotional and behavioural problems was found to be comparable to studies in the West at 12.5% [7]. The same study also found the prevalence of internalising disorders to be more than twice that of externalising disorders, in contrast to studies in the West which showed externalising disorders to be either as common as or in excess of internalising disorders [8-10]. Similarly, Thai and African children were also found to exhibit more over-controlled or internalising behaviour. The Asian culture of promoting self-restraint and emotional control whilst discouraging aggression was hypothesized to explain this difference from Caucasian American children who exhibit more under-controlled or externalising behaviour [11,12].

A small community study that validated a depression scale for adolescents estimates the prevalence of depression to be between 2 and 2.5% [13]. Among those below the age of 14, autism spectrum disorder is the top cause of disease burden while attention deficit hyperactivity disorder and anxiety/depressive disorders rank as the third and fifth leading cause respectively (asthma and low birth weight were the second and fourth causes respectively) [14]. For those in the age group between 15 to 34 years, anxiety/depressive disorders and schizophrenia were the top two leading causes, conferring more healthcare burden than diabetes mellitus and road traffic accident.

Singapore is a highly wired nation with Internet connection penetrating nearly every household [15]. A local survey in Singapore found that 17.1% of secondary school adolescents spent an average of more than 5 hours daily on the Internet and youths [16]. Another study involving primary and secondary school students found the prevalence of pathological gaming to be 8.7% [17], which was much higher than that reported in European adolescents but lower than Hong Kong youths [18,19]. When followed up longitudinally, pathological gamers appeared more likely to develop depression, anxiety, social phobia, and have poorer school performance [20]. At present, the Ministry of Education provides a framework to support schools in delivering cyberwellness programmes within the school curriculum.

Suicide rate is one of the surrogate indicators to measure the mental well-being of a population. Despite young suicides below 20 years being less frequent compared to the elderly, with rates between 2.0 to 2.9 per 100 000 from 1985 to 2004 [21], there was concern about an increasing trend. Although Singapore’s suicide rate among young persons below 24 years approximates the worldwide mean, the gender ratio was equal, unlike many Western nations which often show male preponderance in youth suicide [22]. Jumping from high-rise building is the commonest method for completed suicide [22,23]. For suicide attempts, drug overdose is the commonest method [24]. Academic stress appeared to be significantly associated with suicide among children and adolescents while females were more likely than males to experience preceding relational life stressors [22,25]. Suicide prevention takes a multi-pronged approach with collaborative efforts among government ministries and social services. There have been recent efforts to reduce academic stress among students, such as removal of official ranking of schools by academic performance and removal of T-score reporting for the Primary School Leaving Examinations (a student’s first national examination at Primary Six). Social and emotional learning programmes have also been included in the school curriculum.

### Child and adolescent psychiatry service

The Institute of Mental Health (IMH) is the only public psychiatric hospital in Singapore and is also the largest provider of mental health services. Founded in 1928, child and adolescent mental health service was initially limited to providing custodial care for the severely mentally ill [26]. Child Guidance Clinic (CGC) was set up in 1970 and the number of cases seen grew steadily especially in the 1990s [27]. The inpatient services were started in 1982 and the Department of Child and Adolescent Psychiatry (DCAP) was subsequently formed. While the number of child psychiatrists remained small, the department has grown rapidly, especially over the past 6 years [28]. Today the department’s staff strength is about 130, comprising 12 psychiatrists, psychiatric residents, medical officers, nurses, allied health professionals, administrators and community mental health teams. Currently, the outpatient service (CGC) is located at two sites in Singapore: one within the IMH campus and another located within the city centre at Health Promotion Board building. Treatment interventions offered include medication, psychotherapy, family therapy and academic interventions. The outpatient service is organized into 3 subspecialty clinics. The Mood and Anxiety Clinic offers treatment for children with primarily mood and anxiety disorders. Psychotherapies including cognitive behavioural therapy, interpersonal therapy and dialectical behavioural therapy are mainly provided by the clinical psychologists as well as a few trained psychiatrists. The Neurobehavioural Clinic offers assessment and treatment services for Attention Deficit Hyperactivity Disorder (ADHD), autism spectrum disorders and learning disorders. Treatment programmes include group psycho-education workshops for caregivers, individual behavioural treatment and parent training. The forensic service, also known as Forensic, Rehabilitation, Intervention, Evaluation and Network Development Service (FRIENDS), offers specialist assessment and intervention for children who are victims of abuse or are involved in criminal and antisocial activities. Clinical psychologists within the service are additionally trained in trauma-focused cognitive behavioural therapy. Within the clinic, family therapy is also provided by trained allied health professionals and psychiatrists. The 20-bedded inpatient ward is located within IMH grounds and is run by a multi-disciplinary team comprising psychiatrists, nurses, clinical psychologists, medical social workers, occupational therapists and specialist teachers. The average length of admission for admitted patients for the year 2013 was 14 days, and the top 5 conditions among youths seen in the outpatient and inpatient services for the year 2013 are presented in Table 2.

**Table 2:** Number of patients and top 5 conditions* seen at DCAP (IMH) in 2013

	**Outpatient**	**Inpatient**
No. of		
- New cases or admissions	2,521	129
- Repeat cases or admissions	10,422	102
Top 5 diagnoses	1. Attention deficit hyperactivity disorder	1. Adjustment disorder
	2. No mental illness	2. Depression
	3. Autism spectrum disorder	3. Acute stress reaction
	4. Acute stress reaction	4. Bipolar disorder
	5. Anxiety disorder	5. Psychotic disorder

*These are clinical diagnoses made by the attending psychiatrists based on the International Classification of Diseases or ICD.

Youths may be referred to CGC by doctors, schools, police, other government agencies (e.g. MSF) or as walk-ins. In an unpublished study of all patients referred to the clinic and diagnosed with ADHD in 2002, two-thirds were in lower primary school (median age of 8.0 ± 1.6 years) and were mostly referred by doctors and schools. About half of the patients received medication (mainly methylphenidate, which is the only stimulant medication approved for use in Singapore by the Health Sciences Authority) after a period of follow-up care, likely reflecting clinician practice and initial parental concerns over medication side effects.

Other departments within IMH also provide care for youths below 19, often collaboratively with child psychiatrists. These include the Early Psychosis Intervention Programme (EPIP) and the National Addiction Management Service. EPIP was a service developed in 2001 within IMH which focuses on early detection of psychosis, and subsequent treatment by a multidisciplinary team of psychiatrists, psychologists, case managers, social workers, nurses, and occupational therapists [29]. Over the years, EPIP also focused on increasing awareness of psychosis among the general public, and clinicians in the primary healthcare sector (general practitioners, polyclinic doctors and counsellors) [30]. With evolving practice towards early identification and treatment in psychosis, EPIP established the Support for Wellness Achievement Programme (SWAP) to focus on the assessment and treatment of patients aged 16–30 years with at-risk mental state [31,32]. To improve access to help, EPIP also launched a service within the community called Community Health Assessment Team (CHAT) in 2009 under the auspices of the Ministry of Health. This one-stop mental health centre, located in the downtown shopping belt, provides a drop-in mental health assessment service by a professional team comprising psychiatrists and allied health professionals as well as a range of counselling services for young people between the ages of 16 and 30 years. About 601 youths were referred since its inception by March 2013, and most were self-referred or brought in by families, or referred by counsellors within the community [33].

In addition to IMH, child and adolescent mental health services are also provided at 5 other public hospitals: KKWCH, National University Health System (NUHS), Khoo Teck Puat Hospital, Singapore General Hospital (SGH) and Changi General Hospital (CGH). While the latter two hospitals do assess and manage adolescent patients, their services are however oriented toward treating eating disorders (SGH), adolescent mental health issues and trauma (CGH). To date, SGH is the largest centre for the treatment of eating disorders for adolescents above 13 years of age in Singapore [34]. Among those seeking help, anorexia nervosa appears to affect predominantly Chinese teenage girls compared to other ethnic groups [35-37].

### Community mental health service and schools

Over the decades as Singapore became more developed, attendances at outpatient child psychiatric clinics have also increased. Whether this represents a true increase in incidence, or simply a result of increased child psychiatric services or heightened awareness of mental illnesses leading to increased help-seeking behaviour is not known. Regardless, there is a need to meet this increasing demand and to make child psychiatric services more accessible. Majority of the children referred to the clinic are attending school and spend nearly a third of their daily hours in school or involved in school-related activities. Hence, working with schools is essential. All national schools (primary and secondary schools, junior colleges) have a full or part-time school counsellor who is the main person of contact and a bridge between mental health professionals and school personnel. They have basic counseling skills with some possessing Masters degree in Counselling. Additionally, there are Allied Educators (previously known as Special Needs Officers) who are trained in managing special needs children with mild Dyslexia, ADHD and Autism. Helping these children integrate into mainstream schools and cope with their academic demands are some of the key goals. Sometimes, special school placement might be necessary if the child is unable to integrate back to the school because of their condition.

IMH, NUHS and KKWCH each support a community-based multidisciplinary team (IMH supports 2 teams) of mental health professionals to work directly with school counselors. Each team is called REACH (North, South, East and West, based on school geographical zoning) which stands for “Response, Early Intervention and Assessment in Community mental Health” [38]. By providing consultation liaison service to schools and partnering with trained general practitioners (or family doctors) and voluntary non-government organizations , children and teenagers with suspected mental health conditions and disorders could be assessed in their schools and at their homes if necessary, thereby minimizing disruption to the child’s lessons and reducing the stigma of seeking help [39]. This service thus allows for timely intervention that a psychiatric clinic would ordinarily provide but at a reduced cost. With this model, REACH teams are able to address the mental health issues quickly, alleviate symptoms and reduce morbidity and complications which might arise from delayed treatment.

### Working with community resources

DCAP and REACH teams work closely with schools and community agencies, such as the Singapore Association for Mental Health (SAMH), to help integrate children and adolescents with mental illnesses back to their homes and schools. YouthReach, operated by SAMH, is an activity-based wrap around service for children and adolescents in the process of recovering from their mental illnesses [40]. Comprising a multidisciplinary team, YouthReach performs several tasks including family psychoeducation and support, activity programming and goal-setting for its beneficiaries. One of the key performance indicators is a reduction in re-hospitalization rates. Other voluntary non-government agencies with staff trained in child mental health include Singapore Children’s Society, Methodist Children & Youth Centre, Beyond Social Services and Students Care Service. Beyond collaborative patient care, there are also working relationships in professional training and research with some of these organizations.

### Training in child and adolescent psychiatry

In Asia, there was an overall underdevelopment of CAP postgraduate training systems despite CAP’s recognition as a subspecialty in 12 of 17 of the nations surveyed. The paucity of official guidelines for CAP training was also evident [41]. In Singapore, CAP training is done at 2 main centres: DCAP in IMH and Department of Psychological Medicine in NUHS. Psychiatrist training is administered by the Joint Committee on Specialist Training, the Academy of Medicine and the Division of Graduate Medical Studies of the National University. Prior to 2010, Singapore’s psychiatry training was essentially modelled after the UK system, which was based on apprenticeship and summative assessments. The traditional specialty education began with a 3-year basic specialty training, followed by another 3 years of advanced specialty training. The main training was conducted by senior psychiatrists who were appointed as supervisors by the respective Heads of Departments during the trainees’ hospital rotations. There was a high bar intermediate examination between the basic and advanced years, and an exit examination which the trainee had to pass before becoming a specialist [42].

Common challenges and problems existed within this traditional psychiatry training programme. They included a lack of systematic assessment of core key competencies and continuity of clinical care; poorly organized training schedules and job assignments; large variation in clinical exposure; limited opportunities for feedback on trainee performance; inadequate or inconsistent interactions with senior physicians and supervisors; and a haphazard and arbitrary evaluation framework. The old system simply could not satisfy the expectations of trainee doctors in terms of ensuring a protected 40% of total trainee time for training and preparing them adequately for the high-stake British or local examination, while attempting to meet the demand for clinical service.

With these shortcomings, the Ministry of Health conducted cross-sectional surveys and interviews with specialist and family medicine trainees in 2006–2007 on graduate medical training. The results culminated with the eventual formal introduction of the residency postgraduate training system in 2010, followed by its implementation in different phases across the specialties in medicine. The residency programme thus serves to address and rectify the problems arising from a more traditional training system.

With currently 25 child psychiatrists in both public and private sectors in Singapore and approximately one million children and adolescents under the age of 19 years old, the ratio of child psychiatry to youth population is 1:35,000 or approximately 2.86 per 100,000. This is far from the standards in developed countries, e.g. the national average of 8.67 child and adolescent psychiatrists per 100,000 youths in United States, 2001 [43], even though Singapore ranks among the top ten countries in 2013 with the highest annual GDP per capita [44]. This shortage in specialist manpower was acutely felt in CGC which now typically sees close to 2500 new referrals a year [45] compared to 550 children and families seen in 1980, a four-fold increase. Also, the demand for undergraduate and postgraduate medical school teaching has increased with the opening of Singapore’s third medical school in 2013. There is therefore urgency to recruit, train and nurture junior doctors and residents to become qualified and competent child and adolescent psychiatrists. It is believed that having a strong CAP training curriculum, coupled with higher degrees of professional mentoring, faculty visibility and information accessibility, is necessary to attract residents to consider CAP as a subspecialty [46].

In contrast to the United States of America and other countries with a strong tradition of psychiatry education, there has been no formal CAP specialty training in Singapore. Psychiatrists must complete additional research or clinical fellowships in a reputable child mental health institution or hospital overseas before being considered child-psychiatry trained. In 2014, the Singapore national psychiatry residency programme has developed its first CAP residency training which would span over a one-year period instead of the typical two years [47]. This new residency will take place in the fifth year of residency (called an elective year) and would include clinical rotations in IMH’s DCAP (6 months); pediatric departments in KKWCH or NUHS e.g. Developmental Pediatrics, Adolescent Medicine and Pediatric Neurology (2 months); a Consultation Liaison Psychiatry unit (2 months) and a REACH school mental health team (2 months) with an ongoing continuity clinic in different subspecialized areas, e.g. neurobehavioral, mood & anxiety. Further cross-cultural clinical experience is provided through a clinical or research fellowship (up to a year) in an overseas institution. The Triple Board and integrated training programmes to cross-train in pediatrics and family medicine are not currently offered as local alternatives.

The caseload of a resident is carefully monitored by the resident’s clinical supervisor and by the Programme Director. Direct supervision of cases by a specialist at first visit and at every third visit will also be systematically implemented to ensure professional accountability. The range of clinical cases allows residents to be exposed to all types of childhood and adolescent mental illnesses thus ensuring both breadth and depth of clinical experience. Throughout the clinical attachments, residents are assessed based on observed clinical assessment, 360 degree appraisals and maintenance of their educational portfolios. These assessments would eventually allow for timely intervention, feedback and opportunities for change and improvement. Similarly, each resident is required to provide feedback on the supervision that they have received in their attachments so that the CAP residency programme could be further improved. The residency programme would thus assist in optimizing our human capital by providing quality training and ensuring quality patient care.

### Future challenges

A nation-wide epidemiological study of the prevalence of mental disorders among the young is due, and it may be particularly important to determine the prevalence of autism spectrum disorder due to its associated high burden. A previous study of a group of children diagnosed with autism showed the male to female ratio to be 4.5:1, and the median age at the first consultation to be 41 months [48]. The commonest presenting concern was a delay in the development of speech and language skills in 78% of the children. Although 86% were assessed to have moderate to severe impairment, most improved one year later following centre- or school-based intervention programmes. Early identification and intervention is thus key for developmental disorders like autism. There is a general need to move upstream in the prevention of mental health disorders and develop appropriate programmes for early detection, assessment and treatment of mental illnesses, including in lesser developed fields such as preschool mental health and infant psychiatry. Singapore has performed remarkably well in improving the physical health of our children by reducing infant mortality and increasing life expectancy, both of which now stand amongst the best in the world. The challenge ahead now is to improve the mental and social wellbeing of our children. Such efforts will go beyond the boundaries of traditional medical care to involve multisectoral, multidisciplinary and cross-cultural approaches towards care delivery.

## Abbreviations

ADHD: Attention deficit hyperactivity disorder
CAP: Child and adolescent psychiatry
CGC: Child guidance clinic
CGH: Changi general hospital
DCAP: Department of child & adolescent psychiatry
EPIP: Early psychosis intervention programme
IMH: Institute of mental health
KKWCH: KK women’s and children’s hospital
MSF: Ministry of social and family development
NUHS: National university health system
REACH: Response, early intervention and assessment in community mental health
SAMH: Singapore association for mental health
SGH: Singapore general hospital
SWAP: Support for wellness achievement programme
